# Trajectory Analysis of Orthostatic Hypotension in Parkinson’s Disease: Results From Parkinson’s Progression Markers Initiative Cohort

**DOI:** 10.3389/fnagi.2021.762759

**Published:** 2021-12-20

**Authors:** Kui Chen, Kangshuai Du, Yichen Zhao, Yongzhe Gu, Yanxin Zhao

**Affiliations:** Department of Neurology, Shanghai Tenth People’s Hospital, School of Medicine, Tongji University, Shanghai, China

**Keywords:** orthostatic hypotension, Parkinson’s disease, blood pressure, trajectory analysis, latent class mixed modeling

## Abstract

**Background:** Orthostatic hypotension (OH) in Parkinson’s disease (PD) can lead to falls, impair quality of life, and increase mortality. A trajectory analysis of OH could be useful to predict and prevent the hypotension incidence early.

**Methods:** The longitudinal data of 660 patients with PD with disease duration up to 12 years were extracted from an integrated PD database. We used latent class mixed modeling (LCMM) to identify patient subgroups, demonstrating trajectories of changes in orthostatic blood pressure (BP) over time. The optimal number of subgroups was selected by several criteria including the Bayesian Information Criterion. Baseline information comparison between groups and backward stepwise logistic regression were conducted to define the distinguishing characteristics of these subgroups and to investigate the predictors for BP trajectory.

**Results:** We identified three trajectories for each orthostatic change of systolic blood pressure (ΔSBP), namely, Class 1 (i.e., the increasing class) consisted of 18 participants with low ΔSBP that increased continuously during the follow-up; Class 2 (i.e., the low-stable class) consisted of 610 participants with low ΔSBP that remained low throughout the follow-up; and Class 3 (i.e., the high-stable class) consisted of 32 participants with high ΔSBP at baseline that was relatively stable throughout the follow-up. Several parameters differed among subgroups, but only male sex [odds ratio (OR) = 4.687, 95% confidence interval (CI) = 1.024–21.459], lower supine diastolic blood pressure (DBP) (OR = 0.934, 95% CI = 0.876–0.996), and lower level of total protein at baseline (OR = 0.812, 95% CI = 0.700–0.941) were significant predictors of an increasing ΔSBP trajectory.

**Conclusion:** This study provides new information on the longitudinal development of ΔSBP in patients with PD with distinct trajectories of rapidly increasing, low-stable, and high-stable class. The parameters such as male sex, lower supine DBP, and lower total proteins help to identify the rapidly increasing class.

## Introduction

Orthostatic hypotension (OH), a sustained fall in blood pressure (BP) upon standing, is among the most debilitating manifestations of autonomic dysfunction in Parkinson’s disease (PD). Classical OH is defined as a sustained reduction of at least 20 mmHg of systolic blood pressure (SBP) or 10 mmHg of diastolic blood pressure (DBP) within 3 min of standing or the head-up tilt-table testing (Freeman et al., 2011). Currently, the reported incidence of PD-OH varies from 11.1 to 51.6% (Hommel et al., 2016; Li et al., 2019; Quarracino et al., 2020). The prevalence of OH in PD increases with age and disease duration (Li et al., 2019). OH can impair the blood supply to organs above the heart, such as the brain, resulting in symptoms related to tissue hypoperfusion. The characteristic symptoms include lightheadedness, vertigo, presyncope, and syncope. Symptomatic OH increases the likelihood of falls and has been proven as an independent risk factor of mortality (Masaki et al., 1998; McDonald et al., 2017). There is a significant linear association between the change in systolic BP from supine position to standing and the 4-year mortality rates (Masaki et al., 1998). Furthermore, daily activities and quality of life of patients with PD-OH are significantly compromised relative to patients with PD without OH (Li et al., 2019).

Several confounding variables may influence the extent to which orthostatic BP falls, such as gender, age, hydration, blood glucose, deconditioning, and anemia (Freeman et al., 2011; Perez-Lloret et al., 2012; Li et al., 2019). OH is also independently associated with PD-related parameters, such as the PD phenotype of postural instability and gait disorders (PIGD), lower Mini-Mental State Examination score, longer follow-up time, and higher levodopa equivalent dosage (Hiorth et al., 2019).

However, there have been few studies investigating the longitudinal orthostatic BP changes of patients with PD. BP trajectory could facilitate the prediction of hypotension and hence may be useful in OH prevention. This study was based on the data from the Parkinson’s Progression Markers Initiative (PPMI). The aim of this study was to identify latent subgroups of orthostatic BP trajectories and to investigate the associated factors that influence the different trajectory types. The analysis of orthostatic BP change was based on SBP because the systolic criteria seem to be sufficient to identify 95% of subjects with OH (Fedorowski et al., 2017). In addition, the absolute magnitude of changes in SBP is larger and easier to measure than DBP and as such is more accurate.

## Materials and Methods

### Study Design and Participants

We used data from PPMI, which is an observational, longitudinal, and multicenter study designed to establish the clinical, imaging, and biosample data to define biomarkers of PD progression. The methodology and details of the study assessments are available on the PPMI website^1^. The data were accessed on August 1, 2020. The patients with PD with available BP measurements at two or more visits during a 12-year duration of disease were included. A duration of 12 years was chosen as the upper limit because the majority of participants were patients with *de novo* PD at baseline. Data volumes decrease significantly at longer durations, reducing the reliability of results. From the relationship between disease duration and the number of patients whose BP was measured (Supplementary Figure 1), an upper limit of 12-year duration contained more than 95% BP data (96.43%). Any patient whose primary diagnosis of PD was changed to any other disease during the follow-up was excluded. Each participating PPMI site received ethical approval before study initiation and obtained written informed consent from all participants.

### Clinical Assessments

Parkinson’s disease-related signs and symptoms were assessed with the Movement Disorders Society Unified Parkinson’s Disease Rating Scale (MDS-UPDRS) (Goetz et al., 2008) every year. The motor function was measured with the MDS-UPDRS part 3 (MDS-UPDRS-III) in the off-state. Motor phenotypes were determined as tremor-dominant (TD) phenotype, PIGD phenotype, or indeterminate phenotype, following the classification algorithm proposed by Stebbins et al. (2013). In brief, the PIGD measure includes the five items, namely, freezing, walking and balance in MDS-UPDRS part 2 (MDS-UPDRS-II), gait, freezing of gait, and postural stability in MDS-UPDRS-III; the tremor measure includes the 11 items of tremor in MDS-UPDRS-II, postural tremor, kinetic tremor, rest tremor, and rest constancy in MDS-UPDRS-III. The ratio of tremor score to PIGD score was used to define patients with TD (ratio ≥ 1.15), indeterminate patients (0.9 < ratio < 1.15), and PIGD-dominant patients (ratio ≤ 0.9). The Hoehn and Yahr staging scale (Hoehn and Yahr, 1967) was used for describing how the symptoms of PD progress. The olfactory impairment was measured by the University of Pennsylvania Smell Identification Test (UPSIT). Global cognitive status was assessed using the Montreal Cognitive Assessment. Rapid eye movement sleep behavior disorder (RBD) was screened using the RBD screening questionnaire (RBDSQ). The dosage of PD medications taken was collected and converted to a levodopa equivalent daily dose (LEDD) using the methods described by Tomlinson et al. (2010).

### Vital Signs, Weight, and Height Measurements

Blood pressure (i.e., supine and standing) was measured at every visit. The supine BP was determined after 1–3 min of quiet rest, and the standing pressure was determined after 1–3 min in the standing position. The orthostatic SBP change was calculated as ΔSBP (supine SBP minus standing SBP; note that a positive value represented a drop in SBP with standing). The same was ΔDBP (supine DBP minus standing DBP). Weight and height were collected at the baseline visit and annually or bi-annually according to the visit schedule.

### Clinical Laboratory Tests

Routine clinical laboratory tests consisting of complete blood count and metabolic panel were performed at screening and annually. A central laboratory was implemented to guarantee identical analysis methods, consistent normal ranges, and, thus, common interpretation of laboratory changes. In this research, laboratory parameters possibly related to OH were extracted from all test results, including red blood cell count, hematocrit, hemoglobin, serum glucose, serum sodium, serum potassium, serum chloride, total protein, albumin, creatinine, urea nitrogen, and serum uric acid.

### Statistical Analysis

Continuous variables were expressed as the median and interquartile range (IQR), due to the non-normal data distributions evident upon graphical inspection and application of the Shapiro-Wilk test. Categorical variables were expressed as frequencies and percentages.

We used latent class mixed modeling (LCMM) to model longitudinal ΔSBP. LCMM is a statistical approach that identifies the heterogeneity between patients by classifying them into unobserved subgroups (i.e., latent classes). The time from disease onset (years) was used as the time indicator. The best model was chosen according to (1) Bayesian information criterion (BIC), (2) high mean posterior probability greater than 0.7, (3) sufficient group sizes, and (4) the clinical significance of the models (Louvet et al., 2009; Nagin and Odgers, 2010). To maintain an adequate sample size and clinical significance of each group, we performed models with two to five classes in the current study.

Comparisons of baseline information among subgroups were performed using the Kruskal-Wallis test for quantitative variables and the chi-square test for categorical variables. In the case of significant results, pairwise comparisons were performed, and a Bonferroni correction was applied. Variables with significant differences were further analyzed in the backward stepwise logistic regression of the latent classes.

All data analyses were performed using the SPSS software version 26 (IBM Corp., Armonk, NY, United States) and R software version 4.0.3^2^ with the package “LCMM” for the latent class analysis. A *p*-value of less than 0.05 was considered statistically significant.

## Results

### Demographic and Clinical Characteristics of Participants

A total of 660 participants with available BP at two or more visits during the 12-year duration of disease were included. Characteristics of the study population at the time of entry are presented in Table 1. Continuous variables were expressed as median (i.e., IQR) due to non-normal distribution. The median age at baseline was 63 years (IQR: 55–70), and 58.3% of the population were male. Most patients with PD were newly diagnosed and drug-naive, with a median duration of 2 years (IQR: 1–4) since the symptom onset. Therefore, the majority of patients were within Hoehn and Yahr stage 1 or 2. Based on MDS-UPDRS scores, motor phenotypes were determined as TD (*n* = 439, 66.5%), PIGD (*n* = 154, 23.3%), or indeterminate (*n* = 67, 10.2%). The median of LEDD at baseline was 0 mg (IQR: 0, 405.25). Among all participants, 88 patients (13.4%) presented OH.

**TABLE 1:** Demographical and clinical characteristics of participants.

	Patients (*n* = 660)	Missing, *n*
**Demographics**		
Age, years	63 (55,70)	0
Male, n (%)	385 (58.3%)	0
Weight, kg	78 (67, 88.9)	1
Height, cm	170 (164,178)	1
BMI	26.23 (23.84, 29.67)	1
**PD characteristics**		
Duration, years	2 (1, 4)	0
HY	2 (1,2)	0
MDS-UPDRS-I	6 (3,9)	3
MDS-UPDRS-II	5.5 (3,9)	2
MDS-UPDRS-III	20 (14,27)	0
MDS-UPDRS-IV	0 (0,3)	426
Subtype		0
TD	439 (66.5%)	0
PIGD	154 (23.3%)	0
Indeterminate	67 (10.2%)	0
UPSIT	22 (16,29)	11
MOCA	27 (25,29)	112
RBDSQ	3 (2,5.5)	3
Baseline LEDD, mg	0 (0,405.25)	14
**Blood pressure**		
Supine SBP, mmHg	129 (119, 141)	1
Supine DBP, mmHg	78 (70, 84)	1
Standing SBP, mmHg	124 (115, 138.5)	3
Standing DBP, mmHg	80 (72, 86)	3
ΔSBP, mmHg	3 (−3, 10)	3
ΔDBP, mmHg	−2 (−6, 2)	3
OH, n (%)	88 (13.4%)	3
**Clinical laboratory assessment**		
RBC count, × 10^6^/μl	4.6 (4.35, 4.95)	155
Hematocrit, %	0.41 (0.39, 0.43)	158
Hemoglobin, g/dl	142 (134, 150)	155
Serum glucose, mg/dl	5.4 (5, 5.9)	151
Serum sodium, mmol/L	141 (139, 142)	150
Serum potassium, mmol/L	4.2 (4, 4.4)	151
Serum chloride, mmol/L	103 (101, 104)	150
Total protein, g/dl	70 (68, 73)	150
Albumin, g/dl	41 (40, 43)	151
Creatinine, mg/dl	80 (71, 94)	151
Urea nitrogen, mg/dl	6.1 (5, 7.1)	150
Serum uric acid, mg/dl	303 (250, 357)	150

*BMI, body mass index; DBP, diastolic blood pressure; HY, Hoehn and Yahr staging; LEDD, levodopa equivalent daily dose; MOCA, Montreal Cognitive Assessment; OH, orthostatic hypotension; PIGD, postural instability and gait disorders; RBC, red blood cell; RBDSQ, rapid eye movement sleep behavior disorder screening questionnaire; SBP, systolic blood pressure; TD, tremor dominant; MDS-UPDRS, Unified Parkinson’s Disease Rating Scale; UPSIT, University of Pennsylvania Smell Identification Test; ΔDBP, orthostatic DBP change; ΔSBP, orthostatic SBP change. These values represent the medians, with the interquartile range in parentheses or the number of patients, with percentages in parentheses.*

Considering that long-term PD medication and hypertension may influence ΔSBP, we also calculated the average LEDD and prevalence of hypertension during the follow-up. The median of average LEDD was 473.75 mg (IQR: 300.00–713.24), and a total of 232 patients (35.2%) developed hypertension at baseline or during follow-up.

### Latent Class Analysis of Orthostatic Change of Systolic Blood Pressure

**FIGURE 1:**
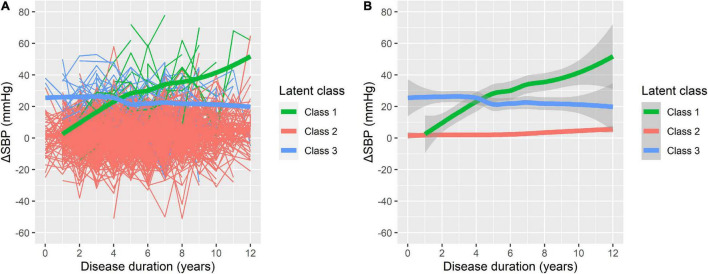
Three latent classes of orthostatic change of systolic blood pressure (ΔSBP) trajectories. **(A)** The raw data for individual patient trajectories in each identified latent class. **(B)** A smoothing of the data represented by bold lines with shaded areas indicating the 95% confidence interval. Class 1: increasing class (*n* = 18, 2.73%); Class 2: low-stable class (*n* = 610, 92.42%); and Class 3: high-stable class (*n* = 32, 4.85%).

On the basis of longitudinal ΔSBP, patients were classified into groups using disease duration as the preferred time indicator. Supplementary Tables 1–3 present the LCMM results of the fitting process. The model of four classes had the lowest BIC; however, there were only three patients (0.45%) in one group of the four classifications. Therefore, the model of three latent classes was regarded as the most appropriate (Figure 1). In this model, Class 1 included 18 participants (2.73%) with low initial ΔSBP that increased consistently during the follow-up (i.e., increasing class). Class 2 included 610 participants (92.42%) with low initial ΔSBP that remained low throughout the follow-up (i.e., low-stable class). Class 3 included 32 participants (4.85%) with higher initial ΔSBP that was relatively stable over time (i.e., high-stable class).

### Comparison of Trajectory Subgroups

The baseline characteristics of participants in different trajectory subgroups are shown in Table 2. BPs at baseline varied significantly among groups as shown in Figure 1. In addition, the three classes differed in multiple variables relating to demographics, PD characteristics, and laboratory tests. In comparison with Class 2, participants in Class 1 were overall older, taller, and had a higher proportion of males, lower levels of total protein, and higher levels of serum potassium. Compared with Class 2, participants in Class 3 were older and taller. Moreover, Class 3 demonstrated a higher incidence of hypertension, higher MDS-UPDRS-III scores, poorer olfaction reflected by UPSIT, and more severe REM sleep disorder represented by RBDSQ. It appeared that older, taller males with severe motor and non-motor symptoms, lower levels of total protein, and higher levels of urea nitrogen tended to develop increasing or high-stable ΔSBP trajectories. However, no significant differences between Class 1 and Class 3 were found except for baseline ΔSBP and serum potassium.

**TABLE 2:** Characteristics of the study population by orthostatic systolic blood pressure change (ΔSBP) trajectory groups.

	Class 1	Class 2	Class 3	*p*	*Post hoc* (Bonferroni adjustment)		
	*N* = 18	*N* = 610	*N* = 32		1 vs. 2	1 vs. 3	2 vs. 3
**Demographics**							
Age, years	69 (64.5, 74)	63 (55, 70)	67 (62.25, 71)	**0.001**	**0.027**	1	**0.024**
Male, *n* (%)	16 (88.9%)	345 (56.6%)	24 (75.0%)	**0.003**	**<0.05**	> 0.05	>0.05
Weight, kg	80.65 (73.48, 90.70)	77.6 (67, 88.75)	79.7 (65.63, 94.1)	0.442	–	–	–
Height, cm	175 (169.5, 180.25)	170 (163, 178)	176.5 (168, 181.5)	**0.003**	**0.067**	1	**0.023**
BMI	25.86 (24.20, 28.26)	26.28 (23.87, 29.70)	25.72 (22.90, 29.64)	0.661	–	–	–
Hypertension	6 (33.3%)	207 (33.9%)	19 (59.4%)	**0.013**	>0.05	>0.05	**<0.05**
**PD characteristics**							
Duration, years	2 (1, 3.5)	2 (1, 4)	2 (1, 2)	0.095	–	–	–
HY	2 (1, 2)	2 (1, 2)	2 (2, 2)	0.114	–	–	–
MDS-UPDRS-I	6 (4, 11)	5 (3, 9)	7 (4, 11)	0.091	–	–	–
MDS-UPDRS-II	8 (5, 9.5)	5 (3, 9)	6 (4, 12)	0.067	–	–	–
MDS-UPDRS-III	20 (15.75, 30.25)	20 (14, 27)	25 (19.25, 30.75)	**0.020**	1	0.805	**0.019**
MDS-UPDRS-IV	0 (0, 2.5)	0 (0, 3)	1 (0, 3.5)	0.818	–	–	–
Subtype				0.536	–	–	–
TD	11 (61.1%)	404 (66.2%)	24 (75.0%)				
PIGD	4 (22.2%)	146 (23.9%)	4 (12.5%)				
Indeterminate	3 (16.7%)	60 (90.8%)	4 (12.5%)				
UPSIT	21.5 (14, 27.25)	23 (16, 29)	16 (12.25, 19.75)	**0.001**	1	0.229	**0.001**
MOCA	28 (26, 28.5)	27 (25, 29)	27 (25.25, 28)	0.523	–	–	–
RBDSQ	5 (3, 7)	3 (2, 5)	5.5 (3, 7)	**0.003**	0.072	1	**0.024**
Baseline LEDD, mg	0 (0, 84.88)	0 (0, 450)	0 (0, 0)	**0.026**	1	0.062	0.425
Average LEDD, mg	416.67 (300.75, 676.83)	480 (300,724.64)	439.29 (296.43,513.79)	0.268	–	–	–
**Blood pressure, mmHg**							
Supine SBP	134.5 (120.25, 142.75)	128 (118, 140)	145.5 (131.5, 150.75)	**0.000**	1	0.175	**<0.001**
Supine DBP	76 (60, 85.75)	78 (70, 83.5)	81 (75.25, 87)	**0.047**	1	0.125	0.06
Standing SBP	120 (110.5, 134.75)	125 (115, 139)	110.5 (102.25, 126)	**0.001**	1	0.365	**0.001**
Standing DBP	76.5 (63, 84.25)	80 (72, 86)	75.5 (70, 80.75)	**0.005**	0.247	1	**0.014**
ΔSBP	10 (4, 14.25)	2 (−4, 10)	29 (17.5, 36.75)	**0.000**	**0.025**	**0.007**	**<0.001**
ΔDBP	0.5 (−1.75, 5.25)	−2 (−7, 2)	9 (0.25, 12)	**0.000**	0.138	0.095	**<0.001**
**Clinical laboratory assessment**							
RBC count, × 10^6^/μl	4.5 (4.3, 4.95)	4.7 (4.4, 5)	4.6 (4.2, 4.9)	0.224	–	–	–
Hematocrit, %	0.41 (0.37, 0.43)	0.41 (0.39, 0.44)	0.41 (0.38, 0.42)	0.083	–	–	–
Hemoglobin, g/dl	143 (126, 149)	142 (134, 150)	141 (130, 150)	0.579	–	–	–
Serum glucose, mg/dl	5.2 (5.1, 5.9)	5.4 (5, 5.9)	5.5 (5.2, 7.3)	0.274	–	–	–
Serum sodium, mmol/L	141 (140.5, 142)	140.0 (139, 142)	141 (139, 142)	0.193	–	–	–
Serum potassium, mmol/L	4.4 (4.15, 4.6)	4.2 (4, 4.4)	4.1 (3.9, 4.4)	**0.040**	0.102	**0.035**	0.582
Serum chloride, mmol/L	103 (102, 104)	103 (101, 104)	103 (101, 105)	0.738	–	–	–
Total protein, g/dl	68 (65, 70.5)	70 (68, 73)	69 (68, 71)	**0.004**	**0.007**	0.382	0.455
Albumin, g/dl	40 (38, 41.5)	41 (40, 43)	40 (40, 42)	0.052	–	–	–
Creatinine, mg/dl	88 (76, 97)	80 (71, 92.25)	80 (71, 101)	0.461	–	–	–
Urea nitrogen, mg/dl	6.5 (5.95, 7.7)	6 (5, 7.1)	6.6 (5.7, 7.9)	**0.008**	0.081	1	0.071
Serum uric acid, mg/dl	339 (306, 378)	299 (250, 357)	303 (268, 345)	0.055	–	–	–

*BMI, body mass index; DBP, diastolic blood pressure; HY, Hoehn and Yahr staging; LEDD, levodopa equivalent daily dose; MOCA, Montreal Cognitive Assessment; OH, orthostatic hypotension; PIGD, postural instability and gait disorders; RBC, red blood cell; RBDSQ, rapid eye movement sleep behavior disorder screening questionnaire; SBP, systolic blood pressure; TD, tremor dominant; MDS-UPDRS, Movement Disorders Society Unified Parkinson’s Disease Rating Scale; UPSIT, University of Pennsylvania Smell Identification Test; ΔDBP, orthostatic DBP change; ΔSBP, orthostatic SBP change.*

*These values represent the medians, with the interquartile range in parentheses or the number of patients, with percentages in parentheses. All significant p-values are highlighted by bold characters.*

### Logistic Regression Analysis of Baseline Predictors for Orthostatic Change of Systolic Blood Pressure Trajectory

**FIGURE 2:**
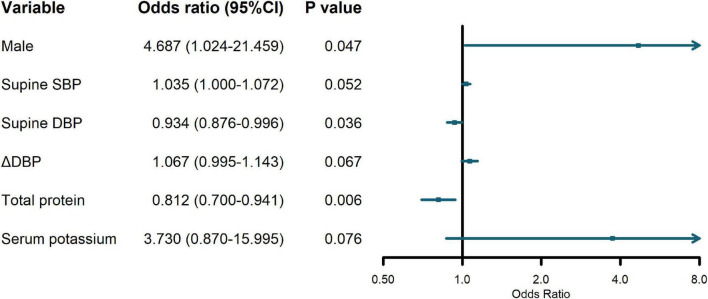
Odds ratios (OR) and 95% confidence interval (CI) of predictor variables on increasing orthostatic change of systolic blood pressure (ΔSBP) trajectory.

Since Class 2 and Class 3 both showed relatively stable trajectories, it was of clinical significance to identify Class 1 with consistently increasing ΔSBP from the two types. In the regression analysis, Class 2 and Class 3 were collapsed into one agreement group, given their similar flat slopes. Due to the interdependent nature of standing BP, supine BP and orthostatic change of BP, only supine BP and BP changes were included in the regression. Regression results revealed that male [odds ratio (OR) = 4.687, 95% confidence interval (CI) = 1.024–21.459], lower supine DBP (OR = 0.934, 95% CI = 0.876—0.996), and lower level of total protein (OR = 0.812, 95% CI = 0.700–0.941) were significant predictors of an increasing ΔSBP trajectory, as presented in Figure 2. The logistic regression model can be considered reliable since the predicted variability resulted in an area under the curve of 0.817 in the ROC analysis (Supplementary Figure 1).

## Discussion

We identified subgroups of ΔSBP trajectory in patients with PD based on a longitudinal analysis of PPMI data. The best fit was identified for a three-class model, reflecting three subgroups of the ΔSBP change. Class 1 was characterized by rapidly increasing ΔSBP. Class 2 displayed a lower level of ΔSBP throughout the follow-up, while Class 3 was characterized by a greater ΔSBP at baseline, which kept relatively stable over time. The main predictors of rapidly increasing ΔSBP in PD were male sex, lower supine DBP, and lower level of total protein at baseline.

Orthostatic hypotension is associated with target organ damage and typically manifests with recurrent syncope and orthostatic intolerance. Previous research has demonstrated that OH increases the risk of falls in patients with PD and is associated with cognitive impairment and greater deterioration in daily living activities, regardless of whether or not OH is symptomatic (Merola et al., 2016, 2018; Romagnolo et al., 2019). Our results show the differences in OH trajectories of patients with PD and in particular highlight those that are worth clinical attention, especially male patients with PD and lower supine DBP and lower total proteins. These patients tend to develop OH and are more prone to falls. As such, this patient group may greatly benefit from an early management strategy that includes advice such as changing positions slowly, particularly upon standing.

In previous studies, there are contradictory results about the association between disease duration and OH. Wüllner et al. (2007) revealed a significant correlation of OH and clinical course through logistic regression analyses. Li et al. (2019) found a difference in disease duration between patients with PD with or without OH; however, disease duration was not found to be a statistically significant influencing factor in PD-OH, with a *p*-value of 0.718 in further logistic regression. In a longitudinal study conducted by Jost and Augustis (2015), there was a lack of a correlation between the severity of OH in PD and disease duration. Nevertheless, in clinical practice, a subset of patients will develop OH during the course of PD. Our findings indicate that, for the majority of patients with PD, ΔSBP remains stable over the disease course. However, in 2.7% of patients, there was a rapidly increasing ΔSBP over the disease course, which may be very difficult to detect in an analysis of the total population.

Clinical variables have been shown to play different roles in cross-sectional studies of PD-OH, as well as in the longitudinal trajectory of PD-OH. In the previous studies, PD-OH has been significantly related to older age, lower body mass index (BMI), more severe motor symptoms, higher LEDD, and higher blood glucose (Perez-Lloret et al., 2012; Nakamura et al., 2017; Li et al., 2019). Similarly, our results showed that older patients with higher MDS-UPDRS-III scores, indicating severe motor symptoms, tend to have higher ΔSBP. In Nakamura et al. (2017), it was emphasized that weight, rather than height, drove the association between BMI and OH. In contrast, however, we found the height of Class 2 to be significantly shorter than that of Class 1 and Class 3, and no association of weight or BMI with OH was identified. Campese et al. (2021) also reported a negative association between classic OH and shorter stature in male patients with PD and considered that the non-neurogenic mechanisms may prevent shorter individuals from BP declines on standing. Considering that some PD medication may be risk factors for OH, we compared the baseline LEDD and the average LEDD during follow-up years among the three classes. No significant difference was found between groups, indicating that LEDD does not change the natural trajectory of ΔSBP. In addition, non-motor scores, BP at baseline, and some laboratory parameters were also different among classes. For example, the patients in the high-stable class had more severe RBD and olfactory dysfunction than those in the low-stable class. It seemed that some patients with PD had remarkable non-motor symptoms at the early stage of PD, indicating the presence of a particular internal phenotypic cluster. It was reported that the combination of olfactory dysfunction, OH, and RBD was associated with a malignant phenotype of PD characterized by more rapid progression of cognitive deficits and postural instability, which could result from the pathological expansion of PD from the olfactory pathway and along fiber tracts in the brain stem simultaneously (Pilotto et al., 2019; Nomura et al., 2020).

In PD, gender is significantly related to multiple motor and non-motor symptoms. In some previous studies, no correlations were identified between OH and gender in patients with PD (Wüllner et al., 2007; Bae et al., 2011). OH was reported for 10% of women and 11% of men in Wüllner et al. (2007), without significant correlation of OH with gender through logistic regression. In contrast, Velseboer et al. (2017) found male sex was associated with the presence of OH in PD. We also found that male sex was one of the predictors of increasing ΔSBP. This finding in patients with PD is distinct relative to the general population, where it is reported that the incidence of OH is higher in women than men (Fedorowski et al., 2009), due to higher estrogen levels, a more active parasympathetic system, lower sympathetic activity, and an attenuated venous distension reflex in women (Cheng et al., 2011; Mansur et al., 2019). Estrogen decreases BP by lowering the plasma renin activity, whereas testosterone decreases BP by increasing the plasma renin activity (Reckelhoff et al., 1998). However, PD is a neurodegenerative disorder with an average onset of approximately 60 years (Obeso et al., 2000), and the median age of patients with PD in this study was 63 years. This stage of life is associated with decreased levels of estrogen and testosterone in women and men, respectively. In addition, older women show a higher sympathetic and lower parasympathetic activity at rest compared with age-matched men (Sachse et al., 2019), providing a possible explanation as to why male patients with PD have a relatively higher incidence of OH.

Another key predictor of increasing OH incidence is supine DBP. Although a decline in diastolic BP during orthostasis may be less relevant in the clinical diagnosis of OH, its potential impact on long-term prognosis requires attention. The diastolic component of BP reflects the real pressure of coronary perfusion as the coronary flow is substantially reduced during systole (Smit et al., 1999). Decreases in DBP may lead to periodic myocardial hypoperfusion and increased orthostatic change in BP. One study has shown that DBP was inversely associated with the risk of myocardial infarction (Fedorowski et al., 2014). Moreover, OH with severe DBP decline is a powerful independent predictor of mortality (Lagro et al., 2012).

Malnutrition is also an important risk factor for OH (Kocyigit et al., 2021). It has been reported that the total protein level is associated with nutritional status in patients with PD (Yang et al., 2020). Kocyigit et al. (2018) revealed that both malnutrition and malnutrition risk were correlated with systolic, but not diastolic, OH. Malnutrition may lead to a reduction in muscle mass and muscle tone (Reijnierse et al., 2015) and bring about impairment in peripheral vasoconstriction, which further causes systolic OH. Our results show that total protein was one of the predictors of increasing ΔSBP. Lower levels of total protein indicated a higher risk for OH. As another parameter for nutritional status, albumin also tended to decrease in Class 1 but without significant difference among groups.

The strengths of our study include a large well-characterized sample, careful screening, comprehensive data collection and processing, and advanced analysis of longitudinal information. However, some methodological limitations ought to be recognized when interpreting the findings. First, the analysis was based on observational data collected at multiple centers, and patients had various confounding factors. Second, there were many other drugs except for PD medication related to OH not included in the analysis. Third, the participants in PPMI were mostly patients with *de novo* PD with short follow-up durations, so, the data for late-stage PD was insufficient. Fourth, the conditions for BP measurement were not rigorously defined. For example, room temperature during BP measurement was not uniform across different centers. Fifth, supine BP was measured after 1–3 min of quiet rest, which was shorter than the standard procedure requirement of 5 min (Lahrmann et al., 2006). Inadequate rest may affect the point measurements but influence the trajectory analysis mildly.

## Conclusion

This study provides new information on the longitudinal development of ΔSBP in patients with PD with distinct trajectories of rapidly increasing, low-stable, and high-stable class. Male sex, lower supine DBP, and lower total proteins helped to identify the class with increasing risk for OH. Further research is needed to discover the biological mechanisms that explain these subgroups and guide future preventive interventions for OH.

## Data Availability Statement

Data used in the preparation of this article were obtained from the Parkinson’s Progression Markers Initiative (PPMI) database (www.ppmi-info.org/access-dataspecimens/download-data). For up-to-date information on the study, visit ppmi-info.org.

## Ethics Statement

Each participating PPMI site received ethical approval before study initiation and written informed consent was obtained from all participants.

## Author Contributions

KC and YaZ designed the study. KC and KD collected the data. KD and YiZ performed the statistical analysis. KC and YG analyzed the results and drafted the manuscript. All authors contributed to the manuscript revision and read and approved the submitted version.

## Conflict of Interest

The authors declare that the research was conducted in the absence of any commercial or financial relationships that could be construed as a potential conflict of interest.

## Publisher’s Note

All claims expressed in this article are solely those of the authors and do not necessarily represent those of their affiliated organizations, or those of the publisher, the editors and the reviewers. Any product that may be evaluated in this article, or claim that may be made by its manufacturer, is not guaranteed or endorsed by the publisher.

## Footnotes

1 PPMI website: http://ppmi-info.org

2 R software version 4.0.3 available at http://www.r-project.org
