# Supplementary material for: Trajectory Analysis of Orthostatic Hypotension in Parkinson’s Disease: Results From Parkinson’s Progression Markers Initiative Cohort
Source: Front Aging Neurosci. 2021 Dec 20;13:762759. doi: 10.3389/fnagi.2021.762759 (PMC8720927; doi:10.3389/fnagi.2021.762759)
Supplement: Supplementary file 1 [file Data_Sheet_1.docx]

Supplementary Material

# Supplementary Figures and Tables

## Supplementary Figures


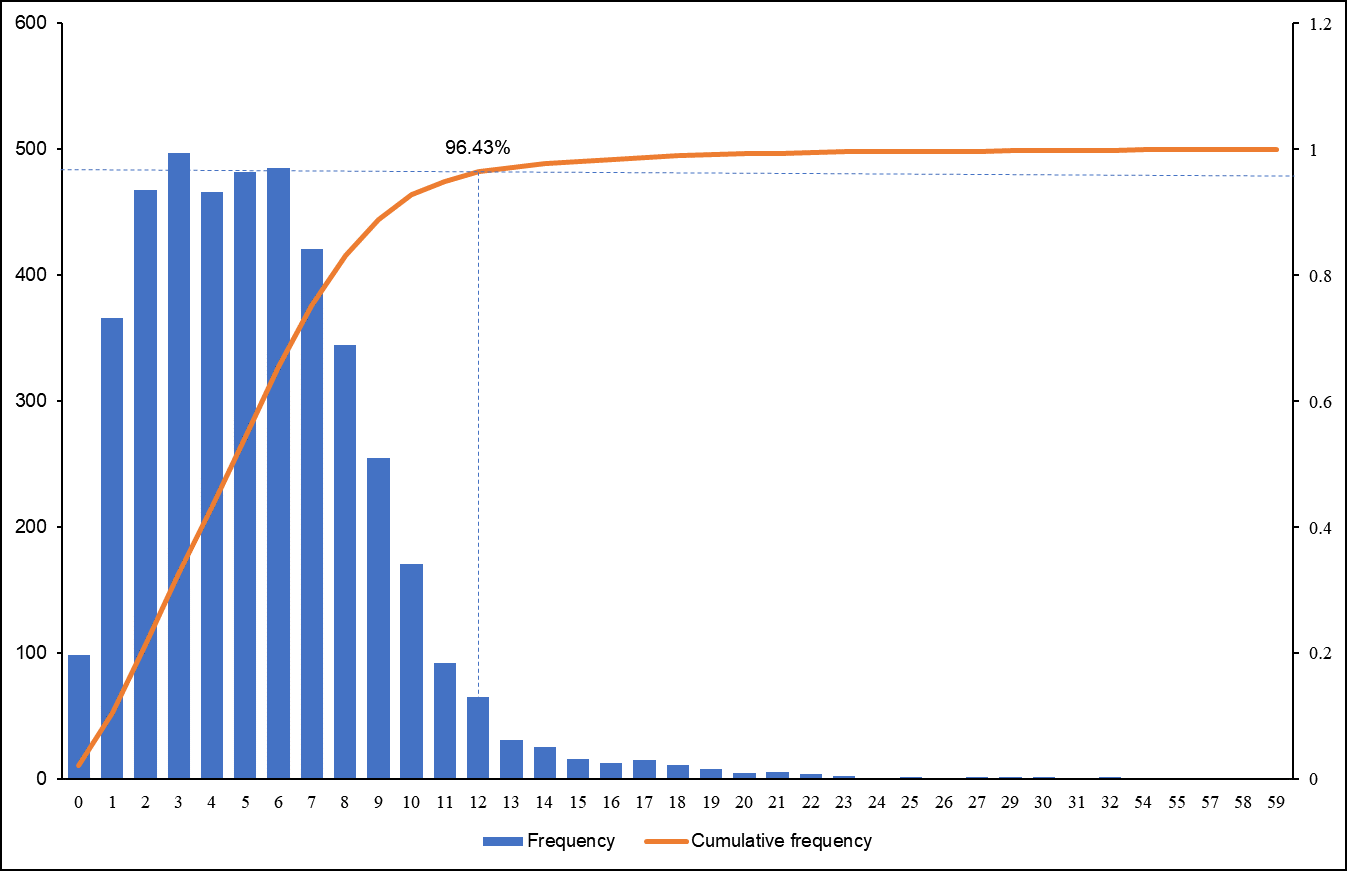


**Figure S1. The relationship between disease duration and the number of patients whose blood pressure was measured from PPMI.** X-axis: disease duration in years. Y-axis: the number of patients whose blood pressure was measured.


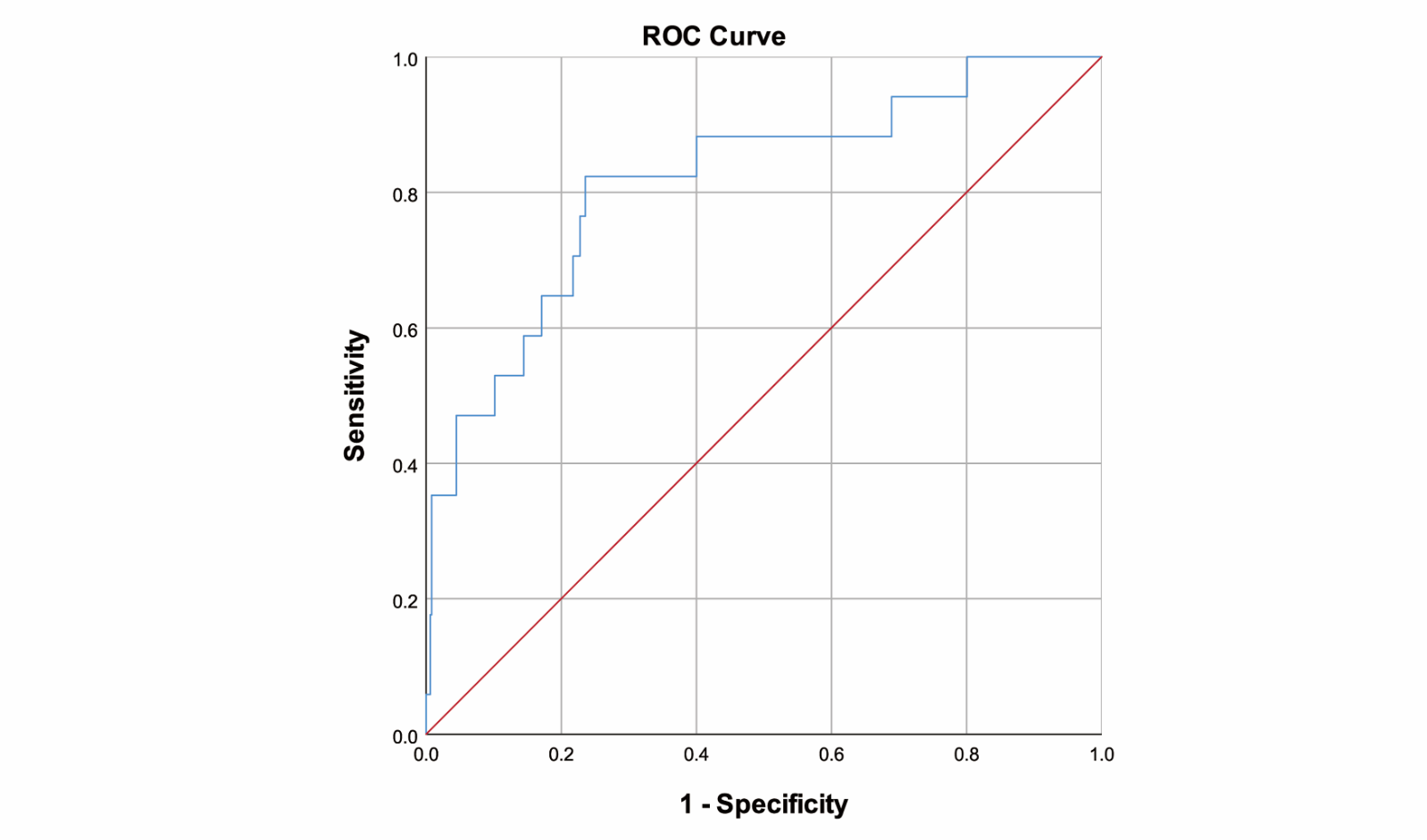
**Figure S2. ROC curve for the binary logistic regression.** The logistic regression model provided a good fit to the data, since the area under the ROC curve was 0.817. ROC, receiver operating characteristics.

## Supplementary Tables

| **Table S1. Model fit evaluation information for each LCMM tested** | | | | | | | |
| --- | --- | --- | --- | --- | --- | --- | --- |
|  | | Maximum log-likelihood | | | BIC | | |
| Two-class LCMM | | -14187.69 | | | 28427.31 | | |
| Three-class LCMM | | -14165.27 | | | 28401.96 | | |
| Four-class LCMM | | -14148.88 | | | 28395.88 | | |
| Five-class LCMM | | -14149.48 | | | 28409.33 | | |
| BIC, Bayesian information criteria; LCMM, latent class mixed model. | | | | | | | |
| **Table S2. Posterior probabilities in each LCMM tested** | | | | | | | |
|  | Class 1 | | Class 2 | Class 3 | | Class 4 | Class 5 |
| Two-class LCMM | 0.8458 | | 0.9854 | - | | - | - |
| Three-class LCMM | 0.804 | | 0.9758 | 0.7041 | | - | - |
| Four-class LCMM | 0.7611 | | 0.9685 | 0.7419 | | 0.8023 | - |
| Five-class LCMM | 0.7567 | | 0.9504 | 0.7263 | | 0.6349 | 0.8028 |

LCMM, latent class mixed model.

| **Table S3. Posterior classification in each LCMM tested** | | | | | |
| --- | --- | --- | --- | --- | --- |
|  | Class 1 | Class 2 | Class 3 | Class 4 | Class 5 |
| Two-class LCMM | 3.94% | 96.06% | - | - | - |
| Three-class LCMM | 2.73% | 92.42% | 4.85% | - | - |
| Four-class LCMM | 2.88% | 91.82% | 4.85% | 0.45% | - |
| Five-class LCMM | 3.03% | 90.61% | 5.00% | 0.91% | 0.45% |

LCMM, latent class mixed model.

| **Table S4. Logistic regression analysis of risk factors associated with ΔSBP trajectory** | | | | | | | | |
| --- | --- | --- | --- | --- | --- | --- | --- | --- |
| Variable | B | SE | Wald | df | *P* | OR | 95% CI | |
|  |  |  |  |  |  |  | Lower | Upper |
| Male | 1.545 | 0.776 | 3.960 | 1 | **0.047** | 4.687 | 1.024 | 21.459 |
| Supine SBP | 0.035 | 0.018 | 3.764 | 1 | 0.052 | 1.035 | 1.000 | 1.072 |
| Supine DBP | -0.069 | 0.033 | 4.379 | 1 | **0.036** | 0.934 | 0.876 | 0.996 |
| ΔDBP | 0.065 | 0.035 | 3.351 | 1 | 0.067 | 1.067 | 0.995 | 1.143 |
| Total protein | -0.209 | 0.075 | 7.654 | 1 | **0.006** | 0.812 | 0.700 | 0.941 |
| Serum potassium | 1.317 | 0.743 | 3.142 | 1 | 0.076 | 3.730 | 0.870 | 15.995 |

Significant P values were highlighted by bold characters.

DBP, diastolic blood pressure; SBP, systolic blood pressure; ΔDBP, orthostatic DBP change; ΔSBP, orthostatic SBP change.
